# A cationic rhodium(I) N-heterocyclic carbene complex isolated as an aqua adduct

**DOI:** 10.1107/S1600536811033125

**Published:** 2011-08-27

**Authors:** Ashley L. Huttenstine, Edward Rajaseelan, Allen G. Oliver, Jeffrey A. Rood

**Affiliations:** a Elizabethtown College, Department of Chemistry and Biochemistry, 1 Alpha Drive, Elizabethtown, PA 17022-2298, USA; b PO Box 1002, Millersville University, Department of Chemistry, Millersville, PA 17551-0302, USA; c University of Notre Dame, Department of Chemistry and Biochemistry, 246 B Nieuwland Science Hall, Notre Dame, IN 46556-5670, USA

## Abstract

The title complex, aqua­[1,3-bis­(2,6-diiso­propyl­phen­yl)imid­az­ol-2-yl­idene](η^4^-cyclo­octa-1,5-diene)rhodium(I) tetra­fluor­ido­borate, [Rh(C_8_H_12_)(C_27_H_36_N_2_)(H_2_O)]BF_4_, exihibits a square-planar geometry around the Rh(I) atom, formed by a bidentate cyclo­octa-1,5-diene (cod) ligand, an N-heterocylcic carbene and an aqua ligand. The complex is cationic and a BF_4_^−^ anion balances the charge. The structure exists as a hydrogen-bonded dimer in the solid state, formed *via* inter­actions between the aqua ligand H atoms and the BF_4_^−^ F atoms.

## Related literature

For the use of N-heterocyclic carbenes (NHCs) in transfer hydrogenation reactions, see: Gnanamgari *et al.* (2006 ▶); Nichol *et al.* (2009 ▶); Hillier *et al.* (2001 ▶). For aqua adducts, see: Feng *et al.* (2010 ▶). For an example of intra­molecular H—F bonding, see: Hobbs *et al.* (2010 ▶). For other NHCs, see: Bappert & Helmchen (2004 ▶); Herrmann *et al.* (2006 ▶); Nichol *et al.* (2010 ▶). For the synthesis, see: Yu *et al.* (2006 ▶). For discussion of complexes with four-coordinate metal atoms, see: Yang *et al.* (2007 ▶).
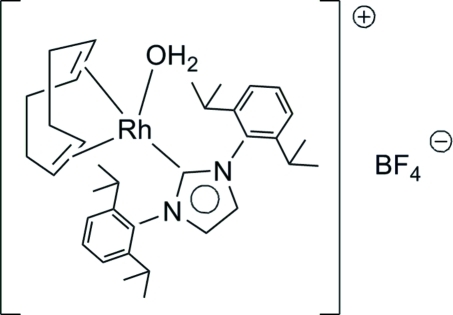


## Experimental

### 

#### Crystal data

[Rh(C_8_H_12_)(C_27_H_36_N_2_)(H_2_O)]BF_4_
*M* *_r_* = 704.49
Triclinic, 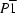

*a* = 11.4351 (4) Å
*b* = 12.2267 (4) Å
*c* = 12.6198 (4) Å
α = 94.103 (2)°
β = 94.081 (2)°
γ = 97.591 (2)°
*V* = 1738.66 (10) Å^3^
*Z* = 2
Mo *K*α radiation
μ = 0.54 mm^−1^
*T* = 150 K
0.28 × 0.25 × 0.18 mm

#### Data collection

Bruker X8 APEXII CCD diffractometer
Absorption correction: multi-scan (*SADABS*; Bruker, 2008 ▶) *T*_min_ = 0.859, *T*_max_ = 0.907
26258 measured reflections
7097 independent reflections
5888 reflections with *I* > 2σ(*I*)
*R*_int_ = 0.038

#### Refinement

*R*[*F* ^2^ > 2σ(*F* ^2^)] = 0.037
*wR*(*F* ^2^) = 0.067
*S* = 1.01
7097 reflections
413 parameters
H atoms treated by a mixture of independent and constrained refinement
Δρ_max_ = 0.49 e Å^−3^
Δρ_min_ = −0.61 e Å^−3^

### 

Data collection: *APEX2* (Bruker, 2008 ▶); cell refinement: *SAINT* (Bruker, 2008 ▶); data reduction: *SAINT*; program(s) used to solve structure: *SHELXS97* (Sheldrick, 2008 ▶); program(s) used to refine structure: *SHELXL97* (Sheldrick, 2008 ▶); molecular graphics: *CrystalMaker* (Palmer, 2009 ▶); software used to prepare material for publication: *enCIFer* (Allen *et al.*, 2004 ▶) and *publCIF* (Westrip, 2010 ▶).

## Supplementary Material

Crystal structure: contains datablock(s) I, global. DOI: 10.1107/S1600536811033125/om2457sup1.cif

Structure factors: contains datablock(s) I. DOI: 10.1107/S1600536811033125/om2457Isup2.hkl

Additional supplementary materials:  crystallographic information; 3D view; checkCIF report

## Figures and Tables

**Table:** 

Rh1—C1	2.046 (2)
Rh1—C33	2.074 (3)
Rh1—C32	2.086 (2)
Rh1—O1	2.117 (2)
Rh1—C29	2.178 (3)
Rh1—C28	2.208 (3)

**Table:** 

C1—Rh1—C33	92.21 (10)
C1—Rh1—C32	94.10 (9)
C33—Rh1—C32	39.31 (11)
C1—Rh1—O1	89.49 (9)
C33—Rh1—O1	159.09 (10)
C32—Rh1—O1	161.19 (10)
C1—Rh1—C29	159.12 (12)
C33—Rh1—C29	97.84 (11)
C32—Rh1—C29	82.49 (11)
O1—Rh1—C29	87.60 (10)
C1—Rh1—C28	164.61 (12)
C33—Rh1—C28	81.68 (11)
C32—Rh1—C28	90.08 (11)
O1—Rh1—C28	91.31 (10)
C29—Rh1—C28	36.23 (12)

**Table 2:** Hydrogen-bond geometry (Å, °)

*D*—H⋯*A*	*D*—H	H⋯*A*	*D*⋯*A*	*D*—H⋯*A*
O1—H1*W*⋯F4	0.80 (3)	1.97 (3)	2.768 (3)	173 (3)
O1—H2*W*⋯F2^i^	0.79 (3)	1.86 (3)	2.644 (3)	175 (3)

Symmetry code: (i) 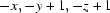.

**Fig. 1.**
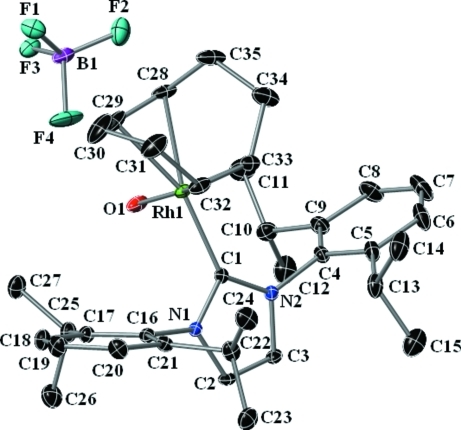
The molecular structure of 1 showing the atom labelling scheme. Displacement ellipsoids are shown at the 30% probablity level.

**Fig. 2.**
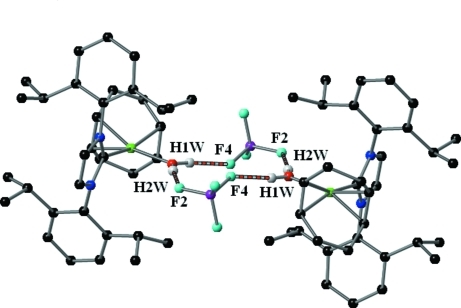
The dimer resulting from H—F interactions in the soild state. The hydrogen bonds are indicated with a dashed-red line.

## supplementary crystallographic information

### Comment

N-heterocyclic carbenes (NHCs) have received tremendous interest in recent times as ligands in catalytic transition metal complexes. An alternative to commonly used phosphines, NHCs provide numerous ways to tune the sterics and electronics of the complex (Herrmann, *et al.*, 2006). Here, we report the crystal structure of the title compound, 1, as a stable aqua adduct that also exhibits H—F bonding interactions in the solid state.

The asymmetric unit of 1 contains a full molecule with the Rh(I) ion in a square planar geometry (Figure 1). The coordination sphere of the Rh(I) is completed through bonds to cycooctadiene, the carbene, and an aqua ligand, creating a complex cation. Charge balance is achieved with a non-coordinating tetrafluoroborate anion.The existence of nearly idealized square planar geometry can be supported using a recently reported metric, τ_4_, for determining molecular shape in four coordinate complexes (Yang, *et al.*, 2007). Here, a τ_4_ value near zero is determined for square planar complexes; however, as the value approaches one, tetrahedral geometry is observed. By evaluation of the bond angles around the central rhodium atom, the τ_4_ parameter was determined to be 0.028 for the title compound.

The bond distances and angles observed in 1 are within the usual ranges for rhodium-carbene [2.046 (2) Å] and rhodium-aqua [2.117 (2) Å] contacts. The O—H distances of the aqua ligand are similar in length [O1—H1W 0.80 (3) Å; O1—H2W 0.79 (4) Å]. The two diisopropylphenyl rings of the carbene ligand are approximately perpendicular to the carbene plane. The carbene atom, C1, deviates from an idealized *sp*^2^ hybridization in that the N1—C1—N2 bond angle is 103.77 (19)°.

Some related cationic rhodium - imidazol-2-ylidene carbene complexes with tetrafluroborate counteranions have been reported (Nichol, *et al.*, 2009, 2010; Bappert, *et al.*, 2004) albeit, with neutral donors other than H_2_O. In the case of 1, presumably due to the steric of the cod and carbene ligands, H_2_O was found to be the only neutral ligand of appropriate size to occupy the fourth coordination site.

The presence of the aqua ligand and tetrafluroborate anion support the formation of a hydrogen-bonded dimer through a center of inversion in the solid state (Figure 2). O—H···F interactions of intermediate strength exist between the aqua ligand and the tetrafluroborate fluorine atoms [H1W—F4 1.968 (4) Å; H2W—F2 1.857 (4) Å]. Interestingly, although an aqua adduct, 1 is not soluble in water likely due to the hydrophobic periphery created by the carbene and cod ligands in the dimer.

In summary, we have reported the crystal structure of a cationic rhodium carbene complex containing an aqua ligand. The structure exists as a hydrogen-bonded dimer in the solid state. Future work aims to investigate the reactivity of this and other similar complexes for various organic transformations.

### Experimental

All chemicals were purchased commercially, except for the neutral rhodium (I) carbene complex, [(cod)Rh(NHC)Cl], which was prepared according to the procedure of Yu, *et al.* (2006). The following manipulations were carried out under an inert nitrogen atmosphere. The cationic compound, [(cod)Rh(NHC)H_2_O]BF_4_, was synthesized by mixing the neutral rhodium compound (0.170 mmol) with water (10 drops) and silver tetrafluoroborate (0.170 mmol) in dichloromethane (20 ml). A yellow solution was obtained after stirring at room temperature for four days along with the formation of a white precipitate. The solution was gravity filtered over Celite and the filtrate was dried *in vacuo* to give a dark orange product (63.33%). X-ray quality crystals were obtained by dissolving the product in a small amount of dichloromethane and layering with pentane. ^1^H NMR (400 MHz, CDCl_3_): *δ* = 1.087 (d, 12H, C*H*_3_-^i^Pr-NHC), 1.399 (d, 12H, C*H*_3_-^i^Pr-NHC), 1.223–1.468 (br, 8H, C*H_2_* -cod), 1.617 (br, H_2_O), 1.890 (br, 2H, C*H*(CH_3_)_2_-^i^Pr-NHC), 2.576 (*sp*, 2H, C*H*(CH_3_)_2_-^i^Pr-NHC), 3.162 (s, 2 H, C*H* -cod), 4.335 (s, 2 H, C*H*-cod), 7.099 (s, 2H, NC*H*), 7.120–7.607(m, 6H, Ar-*H*). ^19^F NMR (376.18 MHz, CDCl_3_): *δ* = -152.105 p.p.m..

### Refinement

Most hydrogen atoms were placed at calculated geometries and allowed to ride on the position of the parent atom. Hydrogen thermal parameters were set to 1.2 times the equivalent isotropic *U* value of the parent atom. C—H distances were constrained as follows: C_aromatic_—H 0.95 Å, CH_3_ 0.98 Å, CH 1.00 Å, and CH_2_ 0.99 Å. The hydrogen atoms of the aqua ligand were found from the difference Fourier map and allowed to freely refine.

### Crystal data

**Table:**

[Rh(C_8_H_12_)(C_27_H_36_N_2_)(H_2_O)]BF_4_	*Z* = 2
*M**_r_* = 704.49	*F*(000) = 736
Triclinic, *P*1	*D*_x_ = 1.346 Mg m^−^^3^
Hall symbol: -P 1	Mo *K*α radiation, λ = 0.71073 Å
*a* = 11.4351 (4) Å	Cell parameters from 8324 reflections
*b* = 12.2267 (4) Å	θ = 2.2–25.1°
*c* = 12.6198 (4) Å	µ = 0.54 mm^−^^1^
α = 94.103 (2)°	*T* = 150 K
β = 94.081 (2)°	Plate, yellow
γ = 97.591 (2)°	0.28 × 0.25 × 0.18 mm
*V* = 1738.66 (10) Å^3^	

### Data collection

**Table:**

Bruker X8 APEXII CCD diffractometer	7097 independent reflections
Radiation source: fine-focus sealed tube	5888 reflections with *I* > 2σ(*I*)
graphite	*R*_int_ = 0.038
Detector resolution: 8.33 pixels mm^-1^	θ_max_ = 26.4°, θ_min_ = 1.6°
φ and ω scans	*h* = −14→14
Absorption correction: multi-scan (*SADABS*; Bruker, 2008)	*k* = −15→15
*T*_min_ = 0.859, *T*_max_ = 0.907	*l* = −14→15
26258 measured reflections	

### Refinement

**Table:**

Refinement on *F*^2^	Primary atom site location: structure-invariant direct methods
Least-squares matrix: full	Secondary atom site location: difference Fourier map
*R*[*F*^2^ > 2σ(*F*^2^)] = 0.037	Hydrogen site location: inferred from neighbouring sites
*wR*(*F*^2^) = 0.067	H atoms treated by a mixture of independent and constrained refinement
*S* = 1.01	*w* = 1/[σ^2^(*F*_o_^2^) + (0.0108*P*)^2^ + 1.7615*P*] where *P* = (*F*_o_^2^ + 2*F*_c_^2^)/3
7097 reflections	(Δ/σ)_max_ = 0.001
413 parameters	Δρ_max_ = 0.49 e Å^−^^3^
0 restraints	Δρ_min_ = −0.61 e Å^−^^3^

### Special details

**Table:**

Geometry. All e.s.d.'s (except the e.s.d. in the dihedral angle between two l.s. planes) are estimated using the full covariance matrix. The cell e.s.d.'s are taken into account individually in the estimation of e.s.d.'s in distances, angles and torsion angles; correlations between e.s.d.'s in cell parameters are only used when they are defined by crystal symmetry. An approximate (isotropic) treatment of cell e.s.d.'s is used for estimating e.s.d.'s involving l.s. planes.
Refinement. Refinement of *F*^2^ against ALL reflections. The weighted *R*-factor *wR* and goodness of fit *S* are based on *F*^2^, conventional *R*-factors *R* are based on *F*, with *F* set to zero for negative *F*^2^. The threshold expression of *F*^2^ > σ(*F*^2^) is used only for calculating *R*-factors(gt) *etc*. and is not relevant to the choice of reflections for refinement. *R*-factors based on *F*^2^ are statistically about twice as large as those based on *F*, and *R*- factors based on ALL data will be even larger.The hydrogen atoms on the water ligand were located from the difference map and their positions were allowed to refine freely.

### Fractional atomic coordinates and isotropic or equivalent isotropic displacement parameters (Å^2^)

**Table:**

	*x*	*y*	*z*	*U*_iso_*/*U*_eq_	
Rh1	0.094766 (17)	0.240311 (16)	0.259517 (18)	0.02419 (6)	
O1	0.0889 (2)	0.36762 (17)	0.38104 (17)	0.0377 (5)	
H1W	0.144 (3)	0.410 (2)	0.409 (2)	0.046 (10)*	
H2W	0.028 (3)	0.378 (2)	0.403 (3)	0.050 (10)*	
N1	0.32568 (17)	0.37772 (15)	0.20142 (16)	0.0219 (4)	
N2	0.36092 (16)	0.26230 (15)	0.31609 (15)	0.0203 (4)	
C1	0.2713 (2)	0.29274 (18)	0.25265 (19)	0.0206 (5)	
C2	0.4452 (2)	0.4000 (2)	0.2356 (2)	0.0270 (6)	
H2	0.5011	0.4559	0.2123	0.032*	
C3	0.4672 (2)	0.3287 (2)	0.3071 (2)	0.0265 (6)	
H3	0.5412	0.3243	0.3446	0.032*	
C4	0.3527 (2)	0.16232 (19)	0.3723 (2)	0.0236 (6)	
C5	0.3193 (2)	0.1656 (2)	0.4764 (2)	0.0281 (6)	
C6	0.3118 (2)	0.0674 (2)	0.5261 (2)	0.0377 (7)	
H6	0.2891	0.0670	0.5971	0.045*	
C7	0.3364 (3)	−0.0296 (2)	0.4750 (2)	0.0418 (7)	
H7	0.3283	−0.0963	0.5097	0.050*	
C8	0.3729 (2)	−0.0288 (2)	0.3732 (2)	0.0374 (7)	
H8	0.3918	−0.0953	0.3390	0.045*	
C9	0.3827 (2)	0.0663 (2)	0.3193 (2)	0.0281 (6)	
C10	0.4301 (2)	0.0665 (2)	0.2099 (2)	0.0353 (7)	
H10	0.4051	0.1316	0.1752	0.042*	
C11	0.3826 (3)	−0.0376 (2)	0.1365 (3)	0.0497 (8)	
H11A	0.4133	−0.1018	0.1645	0.075*	
H11B	0.4081	−0.0283	0.0648	0.075*	
H11C	0.2959	−0.0492	0.1333	0.075*	
C12	0.5661 (3)	0.0812 (3)	0.2204 (3)	0.0476 (8)	
H12A	0.5963	0.1492	0.2654	0.071*	
H12B	0.5955	0.0863	0.1496	0.071*	
H12C	0.5932	0.0176	0.2529	0.071*	
C13	0.3004 (2)	0.2726 (2)	0.5371 (2)	0.0333 (6)	
H13	0.2906	0.3282	0.4838	0.040*	
C14	0.1891 (3)	0.2607 (2)	0.5982 (2)	0.0452 (8)	
H14A	0.1207	0.2284	0.5496	0.068*	
H14B	0.1760	0.3337	0.6285	0.068*	
H14C	0.1995	0.2123	0.6558	0.068*	
C15	0.4102 (3)	0.3169 (3)	0.6117 (2)	0.0516 (8)	
H15A	0.4221	0.2641	0.6649	0.077*	
H15B	0.3993	0.3882	0.6477	0.077*	
H15C	0.4796	0.3270	0.5702	0.077*	
C16	0.2762 (2)	0.4252 (2)	0.1093 (2)	0.0293 (6)	
C17	0.2158 (2)	0.5163 (2)	0.1235 (2)	0.0371 (7)	
C18	0.1762 (3)	0.5617 (3)	0.0312 (3)	0.0586 (10)	
H18	0.1337	0.6232	0.0374	0.070*	
C19	0.1975 (4)	0.5196 (3)	−0.0684 (3)	0.0682 (11)	
H19	0.1708	0.5529	−0.1297	0.082*	
C20	0.2565 (3)	0.4303 (3)	−0.0798 (3)	0.0579 (10)	
H20	0.2693	0.4015	−0.1493	0.069*	
C21	0.2983 (3)	0.3804 (2)	0.0081 (2)	0.0386 (7)	
C22	0.3690 (3)	0.2848 (2)	−0.0069 (2)	0.0430 (8)	
H22	0.3738	0.2491	0.0619	0.052*	
C23	0.3121 (3)	0.1959 (3)	−0.0944 (3)	0.0652 (11)	
H23A	0.3187	0.2251	−0.1643	0.098*	
H23B	0.2284	0.1754	−0.0831	0.098*	
H23C	0.3529	0.1304	−0.0914	0.098*	
C24	0.4954 (3)	0.3263 (3)	−0.0321 (3)	0.0631 (10)	
H24A	0.4933	0.3607	−0.0999	0.095*	
H24B	0.5409	0.2638	−0.0375	0.095*	
H24C	0.5331	0.3809	0.0249	0.095*	
C25	0.1974 (2)	0.5689 (2)	0.2322 (3)	0.0399 (7)	
H25	0.2237	0.5199	0.2867	0.048*	
C26	0.2731 (3)	0.6816 (2)	0.2541 (3)	0.0612 (10)	
H26A	0.3565	0.6733	0.2480	0.092*	
H26B	0.2630	0.7124	0.3262	0.092*	
H26C	0.2484	0.7317	0.2022	0.092*	
C27	0.0672 (3)	0.5797 (3)	0.2451 (3)	0.0773 (12)	
H27A	0.0403	0.6305	0.1950	0.116*	
H27B	0.0587	0.6087	0.3182	0.116*	
H27C	0.0193	0.5069	0.2305	0.116*	
C28	−0.1002 (2)	0.2137 (3)	0.2326 (3)	0.0448 (8)	
H28	−0.1389	0.2796	0.2545	0.054*	
C29	−0.0664 (2)	0.1555 (2)	0.3150 (3)	0.0468 (9)	
H29	−0.0857	0.1860	0.3864	0.056*	
C30	−0.0549 (3)	0.0337 (3)	0.3089 (3)	0.0744 (13)	
H30A	−0.1350	−0.0092	0.3053	0.089*	
H30B	−0.0110	0.0175	0.3751	0.089*	
C31	0.0080 (3)	−0.0051 (2)	0.2139 (3)	0.0581 (10)	
H31A	0.0458	−0.0706	0.2313	0.070*	
H31B	−0.0510	−0.0279	0.1523	0.070*	
C32	0.1017 (2)	0.0846 (2)	0.1830 (2)	0.0362 (7)	
H32	0.1830	0.0629	0.1846	0.043*	
C33	0.0804 (2)	0.1599 (2)	0.1078 (2)	0.0343 (7)	
H33	0.1499	0.1814	0.0662	0.041*	
C34	−0.0368 (3)	0.1690 (3)	0.0490 (3)	0.0586 (10)	
H34A	−0.0544	0.1083	−0.0085	0.070*	
H34B	−0.0307	0.2399	0.0152	0.070*	
C35	−0.1388 (3)	0.1635 (3)	0.1203 (3)	0.0598 (10)	
H35A	−0.2012	0.2033	0.0892	0.072*	
H35B	−0.1732	0.0852	0.1231	0.072*	
B1	0.2301 (3)	0.5986 (3)	0.5597 (3)	0.0379 (8)	
F1	0.29175 (14)	0.70097 (12)	0.55244 (14)	0.0479 (4)	
F2	0.10977 (14)	0.60203 (19)	0.53299 (17)	0.0860 (8)	
F3	0.24800 (18)	0.56624 (15)	0.66043 (15)	0.0629 (5)	
F4	0.26616 (13)	0.52218 (12)	0.48522 (13)	0.0409 (4)	

### Atomic displacement parameters (Å^2^)

**Table:**

	*U*^11^	*U*^22^	*U*^33^	*U*^12^	*U*^13^	*U*^23^
Rh1	0.01932 (10)	0.02176 (11)	0.03008 (12)	−0.00013 (7)	0.00395 (8)	−0.00407 (8)
O1	0.0241 (11)	0.0397 (12)	0.0453 (14)	−0.0032 (10)	0.0089 (11)	−0.0168 (10)
N1	0.0236 (11)	0.0196 (10)	0.0206 (12)	−0.0024 (8)	0.0002 (9)	0.0000 (9)
N2	0.0212 (11)	0.0198 (10)	0.0192 (11)	0.0003 (8)	0.0028 (9)	0.0002 (9)
C1	0.0249 (13)	0.0181 (12)	0.0176 (13)	0.0019 (10)	0.0029 (11)	−0.0049 (10)
C2	0.0212 (13)	0.0268 (14)	0.0305 (16)	−0.0052 (10)	0.0015 (11)	0.0010 (12)
C3	0.0170 (12)	0.0297 (14)	0.0303 (16)	−0.0005 (10)	−0.0022 (11)	−0.0030 (12)
C4	0.0215 (13)	0.0210 (13)	0.0274 (15)	−0.0002 (10)	0.0000 (11)	0.0032 (11)
C5	0.0291 (14)	0.0274 (14)	0.0284 (15)	0.0044 (11)	0.0036 (12)	0.0040 (12)
C6	0.0458 (18)	0.0356 (16)	0.0339 (17)	0.0057 (13)	0.0107 (14)	0.0107 (13)
C7	0.0523 (19)	0.0295 (16)	0.046 (2)	0.0060 (13)	0.0052 (16)	0.0166 (14)
C8	0.0490 (18)	0.0240 (14)	0.0400 (18)	0.0105 (13)	0.0004 (15)	0.0021 (13)
C9	0.0290 (14)	0.0273 (14)	0.0279 (15)	0.0070 (11)	−0.0024 (12)	0.0003 (12)
C10	0.0455 (17)	0.0315 (15)	0.0319 (17)	0.0167 (13)	0.0055 (14)	−0.0009 (13)
C11	0.054 (2)	0.0503 (19)	0.045 (2)	0.0160 (16)	0.0049 (16)	−0.0147 (16)
C12	0.0497 (19)	0.0509 (19)	0.044 (2)	0.0089 (15)	0.0156 (16)	−0.0019 (16)
C13	0.0436 (17)	0.0305 (15)	0.0265 (16)	0.0059 (12)	0.0086 (13)	0.0006 (12)
C14	0.057 (2)	0.0440 (18)	0.0396 (19)	0.0152 (15)	0.0203 (16)	0.0031 (15)
C15	0.063 (2)	0.051 (2)	0.0370 (19)	0.0000 (16)	0.0013 (17)	−0.0064 (16)
C16	0.0331 (15)	0.0273 (14)	0.0246 (15)	−0.0054 (11)	−0.0042 (12)	0.0059 (12)
C17	0.0358 (16)	0.0350 (16)	0.0386 (18)	0.0009 (13)	−0.0069 (14)	0.0082 (14)
C18	0.065 (2)	0.052 (2)	0.059 (3)	0.0150 (17)	−0.0154 (19)	0.0191 (19)
C19	0.093 (3)	0.061 (2)	0.044 (2)	−0.001 (2)	−0.030 (2)	0.0195 (19)
C20	0.095 (3)	0.046 (2)	0.0240 (18)	−0.0153 (19)	−0.0097 (18)	0.0072 (15)
C21	0.0554 (19)	0.0322 (15)	0.0228 (16)	−0.0124 (14)	−0.0009 (14)	0.0038 (13)
C22	0.070 (2)	0.0312 (16)	0.0246 (16)	−0.0068 (15)	0.0121 (15)	−0.0036 (13)
C23	0.093 (3)	0.051 (2)	0.043 (2)	−0.0213 (19)	0.026 (2)	−0.0171 (17)
C24	0.074 (3)	0.047 (2)	0.066 (3)	−0.0060 (18)	0.026 (2)	−0.0096 (18)
C25	0.0368 (16)	0.0337 (16)	0.052 (2)	0.0106 (13)	0.0035 (15)	0.0082 (14)
C26	0.084 (3)	0.0408 (19)	0.057 (2)	0.0006 (18)	0.017 (2)	−0.0047 (17)
C27	0.049 (2)	0.099 (3)	0.094 (3)	0.036 (2)	0.013 (2)	0.031 (3)
C28	0.0180 (14)	0.0496 (18)	0.063 (2)	0.0022 (13)	0.0040 (15)	−0.0201 (18)
C29	0.0329 (17)	0.0409 (18)	0.062 (2)	−0.0142 (14)	0.0287 (16)	−0.0129 (17)
C30	0.083 (3)	0.0376 (19)	0.101 (3)	−0.0153 (18)	0.052 (2)	0.000 (2)
C31	0.055 (2)	0.0267 (16)	0.089 (3)	−0.0075 (14)	0.024 (2)	−0.0086 (17)
C32	0.0294 (15)	0.0243 (14)	0.052 (2)	−0.0008 (11)	0.0074 (14)	−0.0128 (14)
C33	0.0266 (15)	0.0386 (16)	0.0344 (17)	0.0037 (12)	−0.0007 (13)	−0.0145 (14)
C34	0.0424 (19)	0.078 (2)	0.050 (2)	0.0167 (17)	−0.0137 (17)	−0.0255 (19)
C35	0.0266 (16)	0.072 (2)	0.073 (3)	0.0072 (16)	−0.0125 (17)	−0.031 (2)
B1	0.0228 (17)	0.0384 (19)	0.049 (2)	−0.0002 (14)	0.0056 (16)	−0.0154 (17)
F1	0.0514 (10)	0.0286 (9)	0.0596 (12)	0.0034 (8)	−0.0121 (9)	−0.0051 (8)
F2	0.0222 (9)	0.1312 (19)	0.0927 (17)	0.0082 (11)	0.0017 (10)	−0.0643 (15)
F3	0.0855 (15)	0.0551 (12)	0.0446 (12)	−0.0064 (10)	0.0205 (11)	−0.0041 (10)
F4	0.0400 (9)	0.0331 (9)	0.0479 (11)	0.0040 (7)	0.0088 (8)	−0.0118 (8)

### Geometric parameters (Å, °)

**Table:**

Rh1—C1	2.046 (2)	O1—H2W	0.79 (3)
Rh1—C33	2.074 (3)	C2—H2	0.9500
Rh1—C32	2.086 (2)	C3—H3	0.9500
Rh1—O1	2.117 (2)	C6—H6	0.9500
Rh1—C29	2.178 (3)	C7—H7	0.9500
Rh1—C28	2.208 (3)	C8—H8	0.9500
N1—C1	1.366 (3)	C10—H10	1.0000
N1—C2	1.389 (3)	C11—H11A	0.9800
N1—C16	1.449 (3)	C11—H11B	0.9800
N2—C1	1.362 (3)	C11—H11C	0.9800
N2—C3	1.386 (3)	C12—H12A	0.9800
N2—C4	1.452 (3)	C12—H12B	0.9800
C2—C3	1.333 (3)	C12—H12C	0.9800
C4—C5	1.394 (3)	C13—H13	1.0000
C4—C9	1.403 (3)	C14—H14A	0.9800
C5—C6	1.390 (3)	C14—H14B	0.9800
C5—C13	1.516 (3)	C14—H14C	0.9800
C6—C7	1.381 (4)	C15—H15A	0.9800
C7—C8	1.380 (4)	C15—H15B	0.9800
C8—C9	1.384 (4)	C15—H15C	0.9800
C9—C10	1.519 (4)	C18—H18	0.9500
C10—C11	1.533 (4)	C19—H19	0.9500
C10—C12	1.536 (4)	C20—H20	0.9500
C13—C15	1.528 (4)	C22—H22	1.0000
C13—C14	1.530 (4)	C23—H23A	0.9800
C16—C17	1.394 (4)	C23—H23B	0.9800
C16—C21	1.406 (4)	C23—H23C	0.9800
C17—C18	1.398 (4)	C24—H24A	0.9800
C17—C25	1.513 (4)	C24—H24B	0.9800
C18—C19	1.374 (5)	C24—H24C	0.9800
C19—C20	1.362 (5)	C25—H25	1.0000
C20—C21	1.389 (4)	C26—H26A	0.9800
C21—C22	1.516 (4)	C26—H26B	0.9800
C22—C24	1.531 (4)	C26—H26C	0.9800
C22—C23	1.538 (4)	C27—H27A	0.9800
C25—C26	1.524 (4)	C27—H27B	0.9800
C25—C27	1.531 (4)	C27—H27C	0.9800
C28—C29	1.364 (4)	C28—H28	1.0000
C28—C35	1.514 (4)	C29—H29	1.0000
C29—C30	1.509 (4)	C30—H30A	0.9900
C30—C31	1.521 (4)	C30—H30B	0.9900
C31—C32	1.521 (4)	C31—H31A	0.9900
C32—C33	1.399 (4)	C31—H31B	0.9900
C33—C34	1.507 (4)	C32—H32	1.0000
C34—C35	1.520 (4)	C33—H33	1.0000
B1—F1	1.366 (4)	C34—H34A	0.9900
B1—F3	1.368 (4)	C34—H34B	0.9900
B1—F4	1.399 (3)	C35—H35A	0.9900
B1—F2	1.399 (4)	C35—H35B	0.9900
O1—H1W	0.80 (3)		
			
C1—Rh1—C33	92.21 (10)	C12—C10—H10	107.7
C1—Rh1—C32	94.10 (9)	C10—C11—H11A	109.5
C33—Rh1—C32	39.31 (11)	C10—C11—H11B	109.5
C1—Rh1—O1	89.49 (9)	H11A—C11—H11B	109.5
C33—Rh1—O1	159.09 (10)	C10—C11—H11C	109.5
C32—Rh1—O1	161.19 (10)	H11A—C11—H11C	109.5
C1—Rh1—C29	159.12 (12)	H11B—C11—H11C	109.5
C33—Rh1—C29	97.84 (11)	C10—C12—H12A	109.5
C32—Rh1—C29	82.49 (11)	C10—C12—H12B	109.5
O1—Rh1—C29	87.60 (10)	H12A—C12—H12B	109.5
C1—Rh1—C28	164.61 (12)	C10—C12—H12C	109.5
C33—Rh1—C28	81.68 (11)	H12A—C12—H12C	109.5
C32—Rh1—C28	90.08 (11)	H12B—C12—H12C	109.5
O1—Rh1—C28	91.31 (10)	C5—C13—H13	107.6
C29—Rh1—C28	36.23 (12)	C15—C13—H13	107.6
C1—N1—C2	110.6 (2)	C14—C13—H13	107.6
C1—N1—C16	126.3 (2)	C13—C14—H14A	109.5
C2—N1—C16	122.0 (2)	C13—C14—H14B	109.5
C1—N2—C3	111.5 (2)	H14A—C14—H14B	109.5
C1—N2—C4	124.82 (19)	C13—C14—H14C	109.5
C3—N2—C4	122.9 (2)	H14A—C14—H14C	109.5
N2—C1—N1	103.77 (19)	H14B—C14—H14C	109.5
N2—C1—Rh1	125.66 (17)	C13—C15—H15A	109.5
N1—C1—Rh1	129.55 (17)	C13—C15—H15B	109.5
C3—C2—N1	107.5 (2)	H15A—C15—H15B	109.5
C2—C3—N2	106.6 (2)	C13—C15—H15C	109.5
C5—C4—C9	122.7 (2)	H15A—C15—H15C	109.5
C5—C4—N2	119.5 (2)	H15B—C15—H15C	109.5
C9—C4—N2	117.7 (2)	C19—C18—H18	119.2
C6—C5—C4	117.1 (2)	C17—C18—H18	119.2
C6—C5—C13	120.5 (2)	C20—C19—C18	120.4 (3)
C4—C5—C13	122.2 (2)	C20—C19—H19	119.8
C7—C6—C5	121.7 (3)	C18—C19—H19	119.8
C8—C7—C6	119.4 (3)	C19—C20—H20	119.3
C7—C8—C9	121.7 (3)	C21—C20—H20	119.3
C8—C9—C4	117.2 (2)	C21—C22—H22	107.8
C8—C9—C10	120.6 (2)	C24—C22—H22	107.8
C4—C9—C10	122.1 (2)	C23—C22—H22	107.8
C9—C10—C11	113.5 (2)	C22—C23—H23A	109.5
C9—C10—C12	110.4 (2)	C22—C23—H23B	109.5
C11—C10—C12	109.6 (2)	H23A—C23—H23B	109.5
C5—C13—C15	109.8 (2)	C22—C23—H23C	109.5
C5—C13—C14	113.0 (2)	H23A—C23—H23C	109.5
C15—C13—C14	111.0 (2)	H23B—C23—H23C	109.5
C17—C16—C21	122.6 (3)	C22—C24—H24A	109.5
C17—C16—N1	119.7 (2)	C22—C24—H24B	109.5
C21—C16—N1	117.5 (2)	H24A—C24—H24B	109.5
C16—C17—C18	116.6 (3)	C22—C24—H24C	109.5
C16—C17—C25	123.0 (3)	H24A—C24—H24C	109.5
C18—C17—C25	120.3 (3)	H24B—C24—H24C	109.5
C19—C18—C17	121.6 (3)	C17—C25—H25	107.8
C20—C19—C18	120.4 (3)	C26—C25—H25	107.8
C19—C20—C21	121.3 (3)	C27—C25—H25	107.8
C20—C21—C16	117.3 (3)	C25—C26—H26A	109.5
C20—C21—C22	120.1 (3)	C25—C26—H26B	109.5
C16—C21—C22	122.5 (3)	H26A—C26—H26B	109.5
C21—C22—C24	110.8 (2)	C25—C26—H26C	109.5
C21—C22—C23	113.2 (3)	H26A—C26—H26C	109.5
C24—C22—C23	109.2 (2)	H26B—C26—H26C	109.5
C17—C25—C26	110.9 (2)	C25—C27—H27A	109.5
C17—C25—C27	112.3 (3)	C25—C27—H27B	109.5
C26—C25—C27	110.2 (3)	H27A—C27—H27B	109.5
C29—C28—C35	124.6 (3)	C25—C27—H27C	109.5
C29—C28—Rh1	70.69 (16)	H27A—C27—H27C	109.5
C35—C28—Rh1	110.68 (19)	H27B—C27—H27C	109.5
C28—C29—C30	126.3 (3)	C29—C28—H28	114.3
C28—C29—Rh1	73.08 (16)	C35—C28—H28	114.3
C30—C29—Rh1	106.91 (19)	Rh1—C28—H28	114.3
C29—C30—C31	113.9 (3)	C28—C29—H29	114.1
C32—C31—C30	112.1 (2)	C30—C29—H29	114.1
C33—C32—C31	124.0 (3)	Rh1—C29—H29	114.1
C33—C32—Rh1	69.90 (15)	C29—C30—H30A	108.8
C31—C32—Rh1	113.64 (18)	C31—C30—H30A	108.8
C32—C33—C34	126.4 (3)	C29—C30—H30B	108.8
C32—C33—Rh1	70.79 (16)	C31—C30—H30B	108.8
C34—C33—Rh1	111.21 (18)	H30A—C30—H30B	107.7
C33—C34—C35	113.3 (3)	C32—C31—H31A	109.2
C28—C35—C34	112.3 (2)	C30—C31—H31A	109.2
F1—B1—F3	110.1 (3)	C32—C31—H31B	109.2
F1—B1—F4	109.7 (3)	C30—C31—H31B	109.2
F3—B1—F4	110.1 (3)	H31A—C31—H31B	107.9
F1—B1—F2	109.1 (3)	C33—C32—Rh1	69.90 (15)
F3—B1—F2	110.9 (3)	C31—C32—Rh1	113.64 (18)
F4—B1—F2	106.8 (2)	C33—C32—H32	113.9
Rh1—O1—H1W	126 (2)	C31—C32—H32	113.9
Rh1—O1—H2W	120 (2)	Rh1—C32—H32	113.9
H1W—O1—H2W	113 (3)	C32—C33—H33	113.5
C3—C2—H2	126.2	C34—C33—H33	113.5
N1—C2—H2	126.2	Rh1—C33—H33	113.5
C2—C3—N2	106.6 (2)	C33—C34—H34A	108.9
C2—C3—H3	126.7	C35—C34—H34A	108.9
N2—C3—H3	126.7	C33—C34—H34B	108.9
C7—C6—H6	119.1	C35—C34—H34B	108.9
C5—C6—H6	119.1	H34A—C34—H34B	107.7
C8—C7—H7	120.3	C28—C35—H35A	109.1
C6—C7—H7	120.3	C34—C35—H35A	109.1
C7—C8—H8	119.1	C28—C35—H35B	109.1
C9—C8—H8	119.1	C34—C35—H35B	109.1
C9—C10—H10	107.7	H35A—C35—H35B	107.9
C11—C10—H10	107.7		

### Hydrogen-bond geometry (Å, °)

**Table:**

*D*—H···*A*	*D*—H	H···*A*	*D*···*A*	*D*—H···*A*
O1—H1W···F4	0.80 (3)	1.97 (3)	2.768 (3)	173 (3)
O1—H2W···F2^i^	0.79 (3)	1.86 (3)	2.644 (3)	175 (3)

Symmetry codes: (i) −*x*, −*y*+1, −*z*+1.

## References

[bb1] Allen, F. H., Johnson, O., Shields, G. P., Smith, B. R. & Towler, M. (2004). *J. Appl. Cryst.* **37**, 335–338.

[bb2] Bappert, E. & Helmchen, G. (2004). *Synlett*, **10**, 1789–1793.

[bb3] Bruker (2008). *APEX2*, *SAINT* and *SADABS* Bruker AXS Inc., Madison, Wisconsin, USA.

[bb4] Feng, Y., Jiang, B., Boyle, P. A. & Ison, E. A. (2010). *Organometallics*, **29**, 2857–2867.

[bb5] Gnanamgari, D., Moores, A., Rajaseelan, E. & Crabtree, R. H. (2006). *Organometallics*, **26**, 1226–1230.

[bb6] Herrmann, W. A., Schütz, J., Frey, G. D. & Herdtweck, E. (2006). *Organometallics*, **25**, 2437–2448.

[bb7] Hillier, A. C., Lee, H. M., Stevens, E. D. & Nolan, S. P. (2001). *Organometallics*, **20**, 4246–4252.

[bb8] Hobbs, M. G., Knapp, C. J., Welsh, P. T., Borua-Garcia, J., Ziegler, T. & Roesler, R. (2010). *Chem. Eur. J.* **16**, 14520–1433. 10.1002/chem.201001698 20981666

[bb9] Nichol, G. S., Kneebone, J., Anna, L. J. & Rajaseelan, E. (2010). Private communication (deposition No. CCDC785398). CCDC, Cambridge, England.

[bb10] Nichol, G. S., Rajaseelan, J., Anna, L. J. & Rajaseelan, E. (2009). *Eur. J. Inorg. Chem.* pp. 4320–4328.

[bb11] Palmer, D. (2009). *CrystalMaker for Windows* CrystalMaker Software Ltd, Oxfordshire, England.

[bb12] Sheldrick, G. M. (2008). *Acta Cryst.* A**64**, 112–122. 10.1107/S0108767307043930 18156677

[bb13] Westrip, S. P. (2010). *J. Appl. Cryst.* **43**, 920–925.

[bb14] Yang, L., Powell, D. R. & Houser, R. P. (2007). *Dalton Trans.* pp. 955–964. 10.1039/b617136b 17308676

[bb15] Yu, X.-Y., Patrick, B. O. & James, B. R. (2006). *Organometallics*, **25**, 2359–2363.

